# The effects of memory training on behavioral and microstructural plasticity in young and older adults

**DOI:** 10.1002/hbm.23756

**Published:** 2017-08-07

**Authors:** Ann‐Marie Glasø de Lange, Anne Cecilie Sjøli Bråthen, Darius A. Rohani, Håkon Grydeland, Anders M. Fjell, Kristine B. Walhovd

**Affiliations:** ^1^ Center for Lifespan Changes in Brain and Cognition Department of Psychology, University of Oslo Oslo Norway; ^2^ Department of radiology and nuclear medicine Oslo University Hospital Oslo Norway

**Keywords:** plasticity, white matter microstructure, cognitive training, memory, aging

## Abstract

Age differences in human brain plasticity are assumed, but have not been systematically investigated. In this longitudinal study, we investigated changes in white matter (WM) microstructure in response to memory training relative to passive and active control conditions in 183 young and older adults. We hypothesized that (i) only the training group would show improved memory performance and microstructural alterations, (ii) the young adults would show larger memory improvement and a higher degree of microstructural alterations as compared to the older adults, and (iii) changes in memory performance would relate to microstructural alterations. The results showed that memory improvement was specific to the training group, and that both the young and older participants improved their performance. The young group improved their memory to a larger extent compared to the older group. In the older sample, the training group showed less age‐related decline in WM microstructure compared to the control groups, in areas overlapping the corpus callosum, the cortico‐spinal tract, the cingulum bundle, the superior longitudinal fasciculus, and the anterior thalamic radiation. Less microstructural decline was related to a higher degree of memory improvement. Despite individual adaptation securing sufficient task difficulty, no training‐related group differences in microstructure were found in the young adults. The observed divergence of behavioral and microstructural responses to memory training with age is discussed within a supply‐demand framework. The results demonstrate that plasticity is preserved into older age, and that microstructural alterations may be part of a neurobiological substrate for behavioral improvements in older adults. *Hum Brain Mapp 38:5666–5680, 2017*. © **2018 The Authors Human Brain Mapping Published byWiley Periodicals, Inc.**

## INTRODUCTION

The potential for human brain plasticity throughout the lifespan is not yet fully understood [Johansen‐Berg and Duzel, 2016; Walhovd et al., 2015]. As a species, we rely on accumulated experience across decades, suggesting that major neural replacements in the adult brain are not feasible [Rakic, 1985; Walhovd et al., 2015]. Hence, advancing age involves accumulation of changes in forms of wear and tear, and one might expect older adults to exhibit lower capacity for structural brain change relative to younger adults. Possibly counteracting this is how the accumulating brain changes must make the mismatch between functional capacity and environmental demands higher for older adults, as structural plasticity is believed to take place only when demands exceed capacity [Lövdén et al., 2010a]. This paradox, with structural brain differences in aging both constraining plasticity and potentially driving it, calls for a systematic investigation of brain plasticity across age. Thus, the aim of this study was to investigate changes in white matter (WM) microstructure in response to memory training in young and older adults.

Although evidence suggests that brain plasticity is preserved into older age [Burki et al., 2014; Lövdén et al., 2010b; Lustig et al., 2009], a number of studies indicate that young adults tend to show larger cognitive training gains relative to older adults [Baltes and Kliegl, 1992; Burki et al., 2014; Dahlin et al., 2008; Lövdén et al., 2012a; Nyberg et al., 2003]. This is consistent with animal models, where increasing age is associated with a lower magnitude of neuroplastic changes [Blumenfeld‐Katzir et al., 2011; van Praag et al., 2005]. However, studies focusing on human age differences show mixed findings. Juggling exercise has been reported to affect grey matter in both young and older adults [Boyke et al., 2008; Draganski et al., 2004], albeit to a smaller extent in older adults [Boyke et al., 2008]. One study reported that spatial navigation training yielded similar performance gains in young and older adults [Wenger et al., 2012]. While there was evidence of training‐related protection of hippocampal integrity in both young and older adults relative to controls [Lövdén et al., 2012b], training‐related cortical changes were evident in the young adults only [Wenger et al., 2012]. Very few studies have investigated age differences in WM microstructural plasticity in response to training of episodic memory, a cognitive function known as particularly challenging in older age [Nyberg et al., 2012]. Interestingly, one study found that although young adults improved more than older adults on cognitive measures after episodic memory, working memory and perceptual speed training, magnitudes of training‐related WM microstructural plasticity did not differ between the age groups [Lövdén et al., 2010b]. There is ample evidence that memory strategy training, including mnemonic strategies, can be efficient in both young and older adults [Carretti et al., 2011; Cavallini et al., 2003; de Lange et al., 2016; Engvig et al., 2010]. Although this type of training has been shown to influence white matter microstructure in older adults [Engvig et al., 2012], the effects of strategy training on WM microstructural plasticity have not been systematically investigated across age groups.

Further complicating conclusions are the possible effects of factors related to participation, such as general cognitive activity [Gallucci et al., 2009]. As modest cognitive improvement has been observed in active control groups [Legault et al., 2011], the inclusion of such groups is necessary to determine the specific effects of training [Hart et al., 2008; Law et al., 2014]. Hence, a number of training studies include active control groups [Barnes et al., 2013; Fabre et al., 2002; Legault et al., 2011; Lövdén et al., 2010c; Oswald et al., 2006; Schwenk et al., 2010; Suzuki et al., 2012; Zelinski et al., 2011]. However, the inclusion of both active and passive control groups appears to be lacking in studies comparing young and older adults.

Some evidence suggests that the magnitude of structural alterations after training interventions can be linked to the degree of cognitive improvement [Engvig et al., 2010; Hofstetter et al., 2013; Mackey et al., 2012; Scholz et al., 2009]. Conversely, other studies report a lack of associations between cognitive and structural changes [Lövdén et al., 2010b, 2012b, 2013], and it has thus been suggested that the amount of time spent on training may have a larger impact on brain plasticity than improvements in performance [Scholz et al., 2009]. Evidently, there is a need to clarify plastic potential in young versus older adults, and how structural alterations relate to cognitive improvement.

In this study, microstructural changes related to memory training were measured using diffusion tensor imaging (DTI), from which mean diffusivity (MD), fractional anisotropy (FA), radial diffusivity (RD), and axial diffusivity (AD) were derived. DTI measurements reflect the restriction of the water molecules, which can be imposed by microstructure such as myelin, microtubules, and cell membranes [Beaulieu, 2002]. MD represents the mean molecular motion independent of tissue directionality, and is suggested to relate to cellular properties such as size and integrity [Basser, 1995; Pierpaoli et al., 1996]. Evidence suggests that FA is related to restricted molecular motion caused by directionally oriented microstructures such as myelin sheaths and axonal cell membranes [Beaulieu, 2002; Pierpaoli et al., 1996]. AD and RD represent the rate of diffusion along the primary and secondary axes of the diffusion ellipsoid, respectively [Bennett and Madden, 2014]. Although the exact neurobiological underpinnings of diffusion metrics cannot be directly inferred [Wheeler‐Kingshott and Cercignani, 2009], these measures reflect MRI signal changes that may be influenced by alteration in cellular properties [Zatorre et al., 2012].

Memory improvement and changes in DTI metrics were measured across young and older trainers, passive, and active controls. The training group received 10 weeks of memory strategy training aimed at improving serial verbal recollection memory by implementing the mnemonic technique Method of loci (MoL) [Bower, 1970]. The active control group program involved popular scientific topics. The intervention programs were matched to involve similar amounts of cognitive and social engagement. As individual adaptation of task difficulty is considered crucial to evoke plastic responses [Jones et al., 2006; Lövdén et al., 2012a], the memory training was individually adapted for both young and older participants, to continuously place demands above each individual's present level performance. We hypothesized that (i) only the training group would show improved memory performance and alterations in WM microstructure, (ii) that the young adults would show larger memory improvement and a higher degree of WM microstructural alterations relative to the older adults, and (iii) that improvement in memory performance would relate to changes in WM microstructure.

## METHODS AND MATERIALS

### Sample

The sample was drawn from the project *Neurocognitive Plasticity* at the Research Group for Lifespan Changes in Brain and Cognition (LCBC), Department of Psychology, University of Oslo. All procedures were approved by the Regional Ethical Committee of Southern Norway, and written consent was obtained from all participants. Participants were recruited through multiple newspaper and webpage adverts, which ran between one and seven days. All participants were screened with a health interview. Participants were required to be either young or older (in or around their 20s or 70s, respectively) healthy adults, right handed, fluent Norwegian speakers, and have normal or corrected to normal vision and hearing. Exclusion criteria were history of injury or disease known to affect central nervous system (CNS) function, including neurological or psychiatric illness or serious head trauma, being under psychiatric treatment, use of psychoactive drugs known to affect CNS functioning, and magnetic resonance imaging (MRI) contraindications. Moreover, for inclusion in the present study, participants were required to score above 25 on the Mini Mental State Examination (MMSE) [Folstein et al., 1975] and less than 2 standard deviations (SD) below mean on the five minutes delayed recall subtest of the California Verbal Learning Test II (CVLT II) [Delis et al., 2000]. Three individuals in the older group were excluded based on these criteria. All participants further had to achieve an IQ above 85 on the Wechsler Abbreviated Scale of Intelligence (WASI) [Wechsler, 1999]. All scans were evaluated by a neuroradiologist and deemed to be free of significant injuries or conditions. Only participants who completed MR scanning at both baseline and follow‐up in addition to two assessment sessions were included in the current analyses. 15 of the older participants dropped out after the first scanning session (13 in the training group, 1 in the active control group, and 1 in the passive control group). Of the younger participants, 19 dropped out (12 in the training group, 5 in the active control group, and 2 in the passive control group). The reasons included that the participation was too time consuming or that the particular time frame for assessment was inconvenient. At the time of this study, 72 young and 111 older adults—a total of 183 participants—fulfilled the inclusion criteria. Sample demographics for the subjects included are listed in Table 1.

**Table 1:** Sample demographics

	Young adults			Older adults		
	Training group	Active control	Passive control	Training group	Active control	Passive control
	(18F/13M)	(11F/2M)	(13F/15M)	(23F/21M)	(11F/7M)	(33F/16M)
	M ± SD	M ± SD	M ± SD	M ± SD	M ± SD	M ± SD
Age	26.0 ± 3.3	26.6 ± 3.2	26.1 ± 3.0	73.3 ± 2.7	73.5 ± 2.9	73.4 ± 3.2
Edu	15.6 ± 1.8	15.4 ± 2.1	15.8 ± 2.2	15.7 ± 3.1	16.2 ± 2.7	14.3 ± 2.6
MMSE	29.0 ± 1.2	29.5 ± 0.7	29.3 ± 1.1	28.8 ± 1.3	28.2 ± 1.5	28.8 ± 1.1
IQ	110.9 ± 9.9	112.1 ± 5.2	114.6 ± 8.5	122.4 ± 11.1	121.3 ± 5.6	118.4 ± 10.3
CVLT L	61.4 ± 5.5	62.3 ± 9.4	62.8 ± 8.6	47.2 ± 10.8	50.0 ± 10.0	47.0 ± 10.3
CVLT R	13.9 ± 1.7	14.5 ± 2.1	13.9 ± 2.5	10.1 ± 3.5	11.4 ± 3.3	9.9 ± 2.8
MRI interval (days)	77.2 ± 3.9	77.9 ± 1.8	76.7 ± 0.9	75.8 ± 8.3	77.3 ± 1.1	76.6 ± 3.1

The table includes demographics for the included participants measured at baseline. Analysis of variance (Bonferroni corrected) showed no differences between the intervention groups in the young and the older samples, respectively (all *P* values above 0.16, with the exception of a tendency toward a difference in education between the passive and the active control group in the older sample (*P* = 0.051). Across intervention groups, the young adults performed better than the older adults on MMSE (*t* = 2.69, *P* = 0.08), CVLT learning (L) (*t* = 10.99, *P* = 7.82 × 10^−22^) and CVLT recall (R) (*t* = 9.79, *P* = 1.89 × 10^−18^). The older adults showed higher IQ scores than the young adults (*t* = 5.46, *P* = 1.57 × 10^−7^).

The young participants who dropped out performed lower than the rest of the young sample in terms of IQ (mean ± SD for the drop outs = 107.0 ± 10, for the included sample = 113.0 ± 8.7; *t*(71) = 2.3, *P* = 0.02). The group of older participants who dropped out performed lower than the rest of the older sample in terms of IQ (mean ± SD for the drop outs = 114.4 ± 10.5, for the included sample = 120.5 ± 10.2; *t*(109) = 2.2, *P* = 0.03) and CVLT 5 min recall (mean ± SD for the drop outs = 8.3 ± 3.7, for the included sample = 10.2 ± 3.2; *t*(109) = 2.1, *P* = 0.04), and showed a trend toward lower MMS score (mean ± SD for the drop outs = 27.7 ± 1.4, for the included sample = 28.7 ± 1.3; *t*(109) = 2.0, *P* = 0.06).

Lower cognitive performance among dropouts is commonly observed in longitudinal studies, resulting in a selection bias effect toward higher functioning individuals [Salthouse, 2014]. To control for selection bias, we performed a repeated measures analysis of covariance (ANCOVA) to test whether a group of included participants, who matched the participants who dropped out, differed from the rest of the sample in terms of memory improvement. Age and sex were used as covariates. 12 young participants were matched with the young trainers who dropped out on IQ (mean ± SD for the drop outs = 107.2 ± 9.5, for the matched group =107.0 ± 9.0). 13 older participants were matched with the older trainers who dropped out on IQ (mean ± SD for the drop outs = 112.3 ± 9.6, for the matched group = 112.8 ± 9.3). The results showed that the matched groups did not differ from the rest of the training group in terms of memory improvement (*F*(1,28) = 0.38, *P* = 0.5 for the young adults and *F*(1,40) = 0.14, *P* = 0.7 for the older adults).

### Design and Memory Training Program

**Figure 1:**
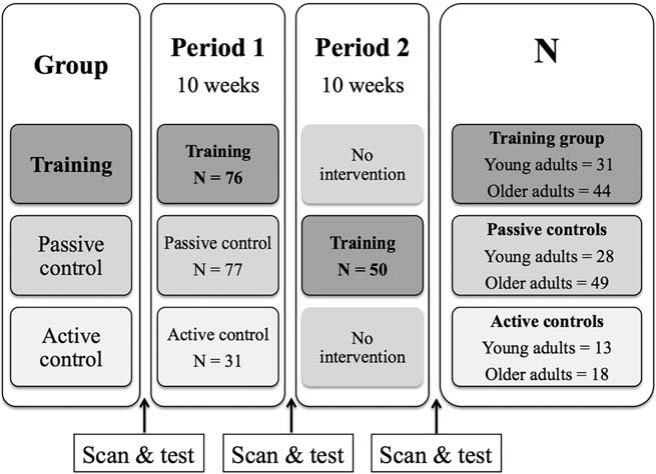
Illustration of the design. *N* represents the number of participants in each group. 39 older adults and 11 young adults received ten weeks of memory training after the initial period as passive controls.

The participants were assigned to one of the three experimental groups depending on registration date. Pools of around 20 participants were recruited at a time, with on‐going data collection for all three conditions simultaneously. Hence, young and old participants from all three experimental groups were scanned and tested interchangeably during the study, reducing the possibility of group differences with regard to the assessment and scanning conditions. All participants completed scanning and cognitive testing at two occasions, with a 10‐week interval between the assessment sessions. Some participants received ten weeks of memory training (older adults: *N* = 44, young adults: *N* = 31), some received 10 weeks of the active control intervention (older adults: *N* = 18, young adults: *N* = 13) and some were scanned and tested before and after 10 weeks as passive controls (older adults: *N* = 49, young adults: *N* = 28). Some of the passive control participants received 10 weeks of memory training after the initial period as passive controls (older adults: *N* = 39, young adults: *N* = 11). Thus, this particular group of participants completed three assessment sessions. These participants were included in a larger training sample used to analyze the statistical relationships between WM microstructural change and memory improvement. The design is illustrated in Figure 1. All participants were examined with MRI and cognitive testing, with a 10‐week interval between each assessment.

The training intervention included practicing the mnemonic technique MoL [Bower, 1970]. The training program included a single course session each week. The first group session included a presentation of the research project, an introduction to the MoL with instructions, and an initial word list task consisting of 15 words. The participants were instructed to memorize the word list by utilizing the MoL [Legge et al., 2012]. The research fellow leading the group session was available for questions, and provided further explanations and repetition of instructions to ensure that all participants were able to utilize the technique. The following weekly group sessions included updating of the strategy, clarification of instructions and a word list task, which was increased by five words each week to ensure a continuous challenge. However, the participants were encouraged to individually adjust the difficulty level, with the aim of adapting a challenging but manageable training level across all the participants. Individual adjustment involved increasing/decreasing the number of words on the tasks to a sufficiently challenging level, performing the tasks within individual time limits and recollection of the word lists in reverse order. Individual adjustments and time limits were discussed in the weekly group sessions. Although time limits and the exact number of words were subject to individual adjustment, all participants completed the training with a weekly increase in number of words. Eight home assignments were sent out weekly, with a minimum requirement that four be completed. The home assignments consisted of word lists with various themes to be memorized using the MoL. The tasks followed the level of difficulty set in the group session the same week, but also included options for individual adjustment in terms of increasing/decreasing the number of words, performing the tasks within stricter time limits and recollection of the word lists in reverse order. The home assignments were completed online. All responses in addition to time spent on the tasks were registered to a database. The number of total tasks completed was on average 74% in the older training group and 45% in the young training group. The active control group program involved popular scientific lectures once a week. Eight home assignments were sent out weekly, with a minimum requirement that four be completed. The home assignments were completed online, and involved tasks related to the weekly popular scientific themes. None of the tasks or lectures in the active control program involved any specific form of memory training. Contact with staff, group meetings and the number of tasks were matched between the training group and the active control group, controlling for the possible effect of these factors on memory performance and WM microstructure. The number of total tasks completed in the active control group was on average 70% for the older adults and 39% for the younger adults. Independent samples *t* tests showed that the number of tasks completed did not differ between the training groups and the active control groups (mean ± SD = 32.7 ± 20.1 for the young training group, 30.8 ± 19.7 for the young active control group, *t*(43) = −0.29, *P* = 0.8, and mean ± SD = 57.6 ± 15.1 for the older training group, 55.5 ± 21.1 for the older active control group, *t*(60) = −0.45, *P* = 0.7). Test sessions and time intervals were held identical for all participants, to ensure that test–retest effects would not differ across the groups.

### Image Acquisition and Analyses

A Siemens Skyra 3 T MRI scanner with a 24‐channel head‐coil was used (Siemens Medical Solutions; Erlangen, Germany). For the current analysis, a diffusion‐weighted echo‐planar imaging (EPI) sequence was applied for each subject (FOV_xy_ = 252 × 256 mm, dimensions = 128 × 130 × 70, voxel size = 1.9626 × 1.9626 mm, slice thickness = 2 mm, repetition time = 9300 ms, echo time = 87 ms). Sixty‐four unique diffusion‐weighted volumes were collected at *b* value = 1000 s mm^−2^ in addition to two non‐diffusion‐weighted (*b* value = 0 s mm^−2^) volumes, one acquired with an opposite *k*‐space traversal direction for the purpose of correcting susceptibility artefacts.

All scan sets were manually checked for gross motion artefacts. The susceptibility‐induced field was estimated using the FSL tool topup [Andersson et al., 2003] and corrected for along with subject motion and eddy current‐induced fields using the eddy tool [Andersson et al., 2012 2012]. Signal dropout caused by subject motion during the diffusion encoding was also detected and corrected [Andersson and Sotiropoulos, 2014]. Each acquired slice was compared with a model‐free prediction, and if the observed signal was statistically different (three SD) from the prediction, it was replaced by the latter. An average of 0.45, 0.42, and 0.45 slices per volume across subjects were replaced in the training group, the passive control group, and the active control group, respectively. The number of slices replaced did not differ between groups (*F*(2, 467) = 0.48, *P* = 0.62). Nonbrain tissue (skull, etc.) was removed using Brain Extraction Tool [Smith, 2002], employing a mask based on the non‐diffusion‐weighted volume. FA images were created by fitting a tensor model to the preprocessed diffusion data using FMRIB's Diffusion Toolbox (FDT) [Behrens et al., 2003].

All participants' FA data were processed with the FSL software package *Tract‐based spatial statistics* (TBSS) [Smith et al., 2006]. The subjects FA images were aligned into a common space using the nonlinear registration tool FNIRT [Andersson et al., 2010], which uses a *b* spline representation of the registration warp field [Rueckert et al., 1999]. Next, the mean FA image was calculated and thinned to create a mean FA skeleton, which represents the centers of all tracts common to the group. The threshold for the mean FA skeleton was set at 0.2, resulting in a mask of 137,832 voxels. Each participant's aligned FA data were then projected onto this skeleton. The nonlinear warps and skeleton projection stages were repeated using the MD, RD, and AD measures. TBSS is documented to be relatively robust to potential partial volume effects (PVE), as it assesses diffusion indices only in the estimated centers of white matter tracts [Berlot et al., 2014; Smith et al., 2006].

### Test of Memory Performance

Memory performance was measured individually using a word list test administrated by a research fellow. Participants were given 5 min to learn as many words as possible in the correct list order, and 10 min to recall the words immediately after the learning trial. The test enabled the MoL to be applied, such that the measure of memory performance was closely related to the utilized technique. To avoid potential ceiling effects, the word list consisted of 100 words. The words in the list differed between time points. All words in the lists were matched on criteria of frequency, complexity, and how easily they were assumed to transfer to visual imagery.

### Statistical Analyses

#### Memory improvement

The total number of words recalled from the word list test was used as the outcome variable for training effects. We have recently shown specific training effects on memory improvement in a group of older adults drawn from the same sample [de Lange et al., 2016]. To examine the group differences in memory improvement for both young and older adults, a repeated measures ANCOVA was conducted on the memory scores (number of words correctly memorized) at baseline and follow‐up (time point 2), using age, sex, and baseline memory performance as covariates. Greenhouse–Geisser corrections for violation of sphericity were used. Additional repeated measures ANCOVAs were run for the training group, active control group, and passive control group separately, testing the change in memory performance from baseline to follow‐up. Effect sizes were calculated using Cohen's d, measuring the difference between the mean change in memory performance in the training group and the control group, divided by the pooled standard deviation [Cohen, 1988]. An independent samples *t* test was performed to compare the number of tasks completed in the training group and the active control group.

#### Group differences in WM microstructural changes

To investigate group differences in mean WM change, we performed general linear model (GLM) analyses on the mean of the skeletonized tensor‐derived values, using age, sex, motion, and baseline WM values as covariates. Motion was estimated as the mean of the average root mean square displacement value across each diffusion‐weighted volume derived from the eddy procedure [Andersson and Sotiropoulos, 2015].

We then performed voxel‐wise GLMs using the values from follow‐up as the dependent variable, and the values from baseline as a per‐voxel regressor, testing the differences between the intervention groups within the young and the older sample, respectively. Permutation‐based statistics with threshold‐free cluster enhancement [Smith and Nichols, 2009] were performed with 5000 permutations [Nichols and Holmes, 2002], correcting for multiple comparisons across space, as implemented in *randomise*, part of FSL [Winkler et al., 2014]. The significance threshold was set at *P* < 0.05, as for all subsequent analyses.

#### Relationships between memory improvement and microstructural changes

To investigate relationships between change in WM microstructure and change in memory performance, all participants who completed the training program were included (*N* = 126 participants, 43 young adults and 83 older adults), as illustrated in Figure 1. Voxel‐wise GLMs were performed on the full skeleton using the values from the MRI assessment after training as the dependent variable, and the values from the assessment before training as a per‐voxel regressor. Permutation‐based statistics were performed with 5000 permutations, as implemented in *randomise*. Age, sex, and motion were used as covariates. Improvement in memory performance was measured by standardized residuals. This measure determines whether changes from baseline to follow‐up are large with respect to the group mean and SD. Standardized residuals were calculated from a linear regression analysis, using memory performance at follow‐up as the dependent variable and memory performance at baseline as the independent variable.

## RESULTS

### Memory Improvement

**Figure 2:**
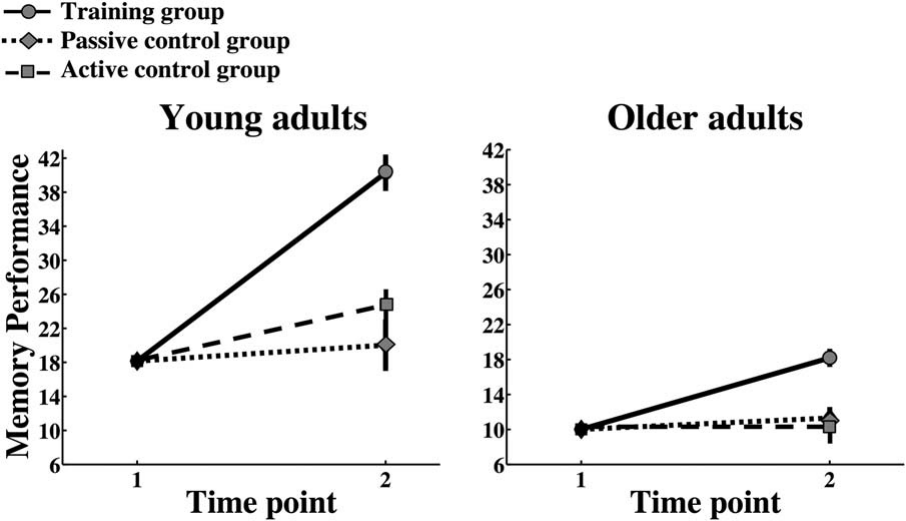
Memory improvement measured by the word list consisting of 100 words is shown for the young and older intervention groups. Memory scores are shown on the *Y*‐axis.

Memory improvement is shown in Figure 2, which includes the present results on group differences in the young sample in addition to group differences within the older sample, as previously published in de Lange et al. [2016]. The analysis showed an interaction between group (training, active control, and passive control) and time (baseline and follow‐up) when including both young and older adults (*F*(2,175) = 56.4, *P* = 1.2 × 10^−19^). Pairwise comparisons (bonferroni corrected) showed that the group receiving memory training improved more than the control groups (training group versus active control group: mean difference = 6.0, *P* = 1.9 × 10^−10^, training group versus passive control group: mean difference = 6.5, *P* = 2.3 × 10^−18^). No differences were found between the active and passive control groups (mean difference = 0.5, *P* = 1.0). The same analysis revealed an interaction between age and change (*F*(1,177) = 58.3, *P* = 1.3 × 10^−12^). Independent samples *t* tests using the relative difference in memory performance (performance at follow‐up minus baseline performance divided by baseline performance) showed that the young training group improved more that the older training group (*t*(74) = 3.1, *P* = 0.002). Separate analyses confirmed significant group interactions within each age group (older adults: *F*(2,102) = 22.4, *P* = 8.1 × 10^−9^; young adults: *F*(2,64) = 36.2, *P* = 2.2 × 10^−11^). Repeated measures tests of within subjects effects showed that the group who received memory training improved from baseline to follow‐up (*F*(1,71) = 75.5, *P* = 7.9 × 10^−13^). None of the control groups improved significantly from baseline to follow‐up. The memory improvement in the training group, as adjusted for the improvement in the control groups, showed an effect size of 1.95 in the young adults and 1.17 in the older adults, which can be regarded as very large and large effect sizes, respectively [Cohen, 1988; Sawilowsky, 2009].

To control for a possible lack of power in the control groups due to smaller sample sizes, we performed a power analysis using G*Power [Faul et al., 2007]. The effects size measured within the training group was *f* = 0.7701, corresponding to a power of 0.96, with 9 subjects required to detect an effect given the *f* value. Thus, the lack of training effects in the control groups, consisting of 77 and 31 participants, respectively, was unlikely to be caused by a power issue.

As this article focuses on training effects on white matter microstructure, the active and the passive control groups were merged into one larger control group on the basis of showing no improvement in memory after 10 weeks. This merged control group was used in further statistical analyses.

### Group Differences in Microstructural Changes

**Figure 3:**
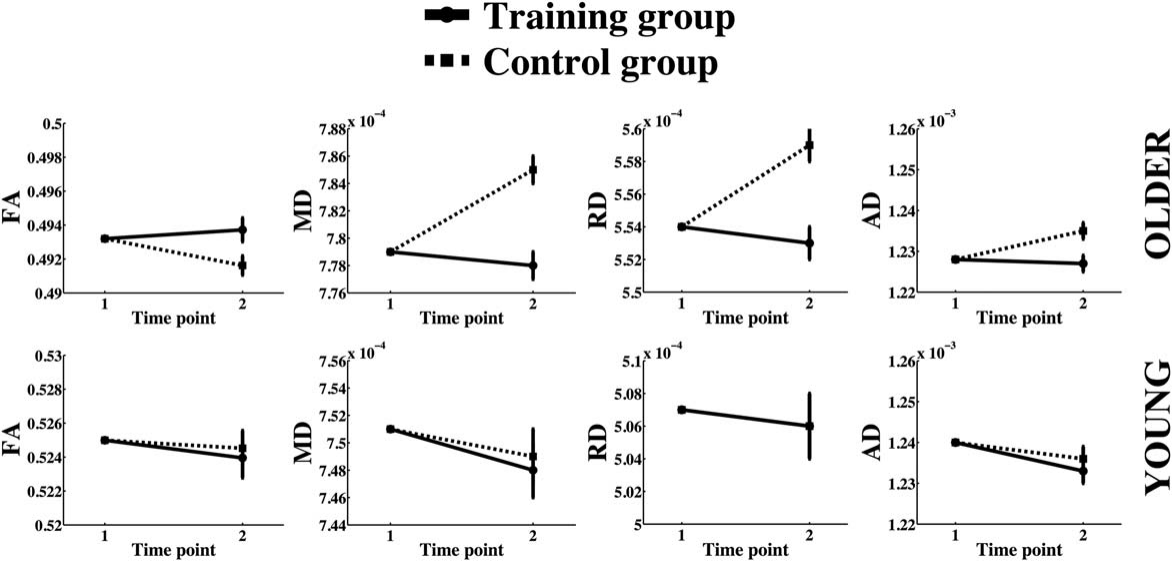
Group differences in microstructural changes are plotted separately for young and older adults. The means of the skeletonized diffusion metrics are shown on the *Y*‐axis. The axis ranges are of equal size for young and older adults, but the values vary due to age differences in diffusion metrics.

The group differences in mean WM change are shown in Figure 3. When including both young and older adults, the analysis of group differences in mean WM change showed an interaction between group (training and control) and time (baseline and follow‐up) in MD (*F*(1,176) = 6.8, *P* = 0.01), RD (*F*(1,176) = 6.7, *P* = 0.01), and AD (*F*(1,176) = 6.4, *P* = 0.01). The control group increased more in MD, RD, and AD relative to the training group. No group differences were found in FA change. The same analysis revealed an interaction between age and change in MD, RD, and AD (*F*(1,176) = 19.3, *P* = 1.9 × 10^−5^, *F*(1,176) = 15.2, *P* = 1.4 × 10^−4^, and *F*(1,176) = 6.3, *P* = 0.01, respectively). As the group of older adults showed higher average IQ than the young adults, the analysis was repeated with the inclusion of IQ as a covariate. IQ did not influence the results of the group differences, and no interactions were found between IQ and WM microstructural changes (MD: *F*(1,177) = 2.1, *P* = 0.14; RD: *F*(1,177) = 1.6, *P =* 0.21; AD: *F*(1,177) = 2.3, *P* = 0.13).

To control for possible effects related to WM lesions, which is known to increase in frequency with older age [Leritz et al., 2014], we performed follow‐up analyses using repeated measures ANCOVA controlling for WM hypointensities in addition to age, sex, motion, and baseline WM values. WM hypointensities were derived for each subject using the FreeSurfer (http://surfer.nmr.mgh.harvard.edu/) probabilistic procedure [Fischl et al., 2002, 2004a, 2004b). This procedure has demonstrated sensitivity in measurements of WM lesions in older adults [Leritz et al., 2014; Salat et al., 2010]. The results showed that WM hypointensities did not affect the results of the group analyses reported above when included as a covariate. No interactions were found between hypointensities and change in MD, RD, and AD (*F*(1,175) = 0.4, *P* = 0.6; *F*(1,175) = 1.1, *P* = 0.3; and *F*(1,175) = 0.1, *P* = 0.7, respectively).

Separate voxel wise analyses on the older groups showed that the control group decreased more than the training group in FA in 1.6% of the voxels (peak *P* value = 0.02) and increased more in MD, RD, and AD from baseline to follow‐up relative to the training group in 8.7%, 14.6%, and 6.8% of the voxels, respectively (*P* < 0.05, peak *P* value = 0.02, 0.02, and 0.02), in areas overlapping the corpus callosum, the corticospinal tract, the cingulum bundle, the superior longitudinal fasciculus (temporal part), and the anterior thalamic radiation. As a follow‐up analysis, GLMs were performed on the difference maps of FA, MD, RD, and AD, using difference scores in memory performance (follow‐up minus baseline). Age, sex, and motion were used as covariates. The results showed that the control group decreased more than the training group in FA in 9% of the voxels (peak *P* value = 0.02) and increased more in MD, RD, and AD from baseline to follow‐up relative to the training group in 19.8%, 21.1%, and 15.5% of the voxels, respectively (*P* < 0.05, peak *P* value = 7.6 × 10^−3^, 7.4 × 10^−3^, and 0.02), in areas overlapping those of the analyses using standardized residuals.

**Figure 4:**
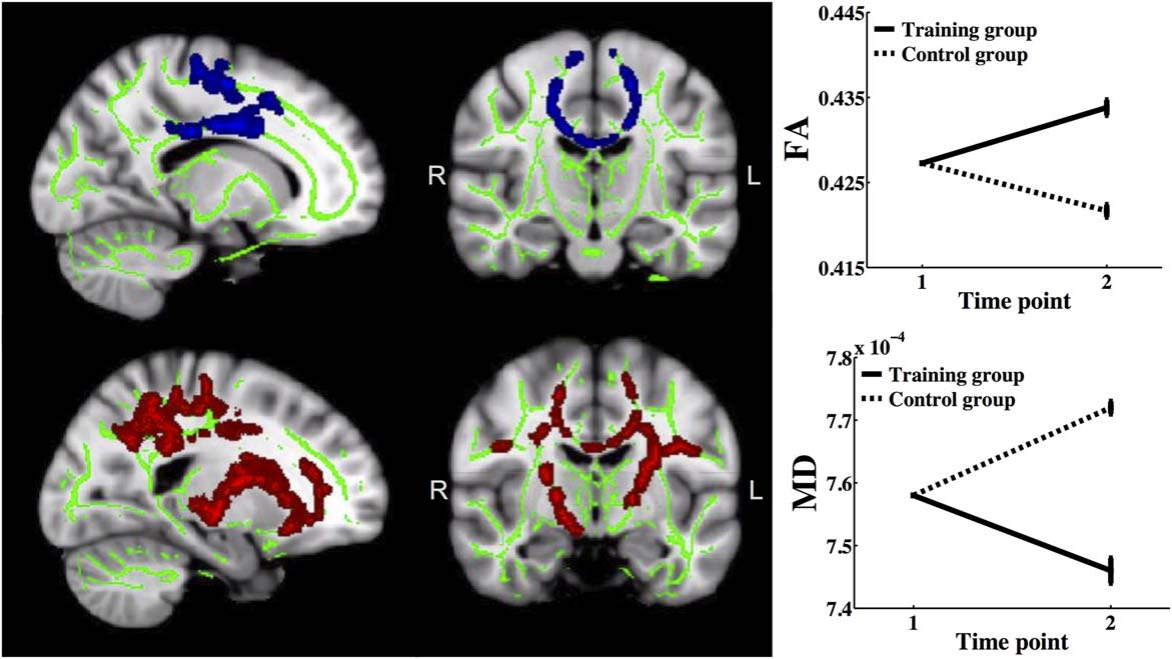
Areas showing group differences in microstructural changes in the older sample. Sagittal and coronal views of Talairach coordinates *x* = 105, *y* = 110, *z* = 112 for fractional anisotropy (FA) and *x* = 110, *y* = 117, *z* = 112 for mean diffusivity (MD), overlaid on the mean FA skeleton (green) and the standard MNI152 T_1_ 1 mm^3^ brain template. The results are thresholded at *P* < 0.05 and corrected for multiple comparisons. Significant areas are dilated for illustrative purposes. The plots show the mean values within the respective areas of group differences in MD and FA.

No differences were found between the training and control groups in the young sample. The group differences in WM change in the older adults are shown in Figure 4.

### Relationship Between Memory Improvement and Microstructural Changes

**Figure 5:**
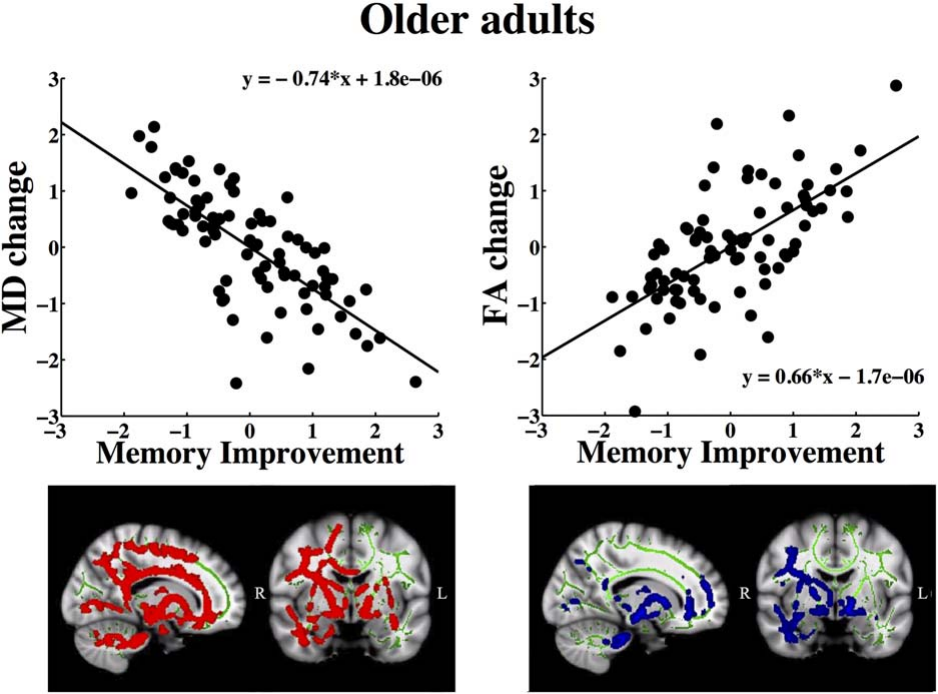
Areas showing relationships between memory improvement and microstructural changes in the older sample. Sagittal and coronal views of Talairach coordinates *x* = 74, *y* = 120, *z* = 85, overlaid on the mean FA skeleton (green) and the standard MNI152 T_1_ 1 mm^3^ brain template. The results are thresholded at *P* < 0.05 and corrected for multiple comparisons. Significant areas are dilated for illustrative purposes. The plots show the relationships between MD and FA change and memory improvement measured by standardized residuals.

The relationships between memory improvement and change in FA and MD are shown in Figure 5. A positive relationship was found between change in FA and memory improvement in the older adults in 12.87% of the voxels (peak *P* value = 7.0 × 10^−3^). Changes in MD, RD, and AD correlated negatively with memory improvement in 25.6%, 24.1%, and 24.1% of the voxels, respectively (*P* < 0.05, peak *P* value = 1.5 × 10^−3^, 1.8 × 10^−3^, and 1.5 × 10^−3^). Changes in RD and AD occurred in areas overlapping those of MD. As a follow‐up analysis, GLMs were performed on memory improvement and FA and MD change using difference scores (follow‐up minus baseline). Age, sex, and motion were used as covariates. The results showed a tendency for a positive correlation between difference in memory performance and FA change (peak *P* value = 0.06), while no relationship was found between difference in memory performance and MD change (peak *P* value = 0.14).

No relationships were found between microstructural changes and memory change in the older control group. In the younger adults, no relationships were found between change in WM microstructure and memory improvement.

### Relationships Between Number of Tasks, Memory Improvement, and Microstructural Changes

As a follow‐up analysis, we tested whether number of tasks completed during the training period was related to (a) memory improvement and (b) changes in WM microstructure. Age and sex were used as covariates. An independent samples *t* test showed that the older participants completed more tasks (mean ± SD = 57.41 ± 14.13) during the training period relative to the young group (mean ± SD = 34.67 ± 18.60, *t*(74) = 7.66, *P* = 1.0 × 10^−3^). However, Pearson correlation analyses showed that the number of tasks completed during the training period did not correlate with either memory improvement or changes in WM microstructure (when corrected for memory improvement) in any of the age groups.

### Utilization of the MoL

The use of a mental travel route in the MoL enables the words in the word list tasks to be recalled in a specific order [Legge et al., 2012]. As an indication of whether the participants successfully applied the MoL, we investigated the increase in performance on word recollection in the specific order corresponding to the word list test used as the outcome measure. First, the mean percentage change in the number of words provided in the correct order [(time point 2 performance − baseline performance)/baseline performance] was calculated in the young and the older control groups, as an indication of practice effects [Bartels et al., 2010]. To identify potential outliers, regression analyses were run with performance at time point 2 as the dependent variable and performance at baseline as the independent variable. Outliers were identified by studentized deleted residuals (SDR) exceeding ± 3. While no outliers were found in the young control group, two outliers were identified in the older control group (SDR = 3.58 and 3.53). The mean improvement (excluding the outliers) was 0.40 (SD = 0.95) in the young control group, and 0.60 (SD = 1.17) in the older control group. We then identified the percentage of the participants in the training group who improved their performance to a higher extent than the mean of the control groups. 84.4% of the participants in the young training group and 72.1% in the older training group showed an increase in performance that exceeded the means of the young and the older control groups, respectively.

## DISCUSSION

This study aimed to investigate changes in WM microstructure in response to 10 weeks of memory strategy training in young and older adults. Overall, the results demonstrated that only the group that received the memory training intervention, rather than either the active or the passive control condition, showed significant improvement in memory performance. In the older adults, the training group showed less degree of age‐related decline in WM microstructure in comparison to the control group, indicating that episodic memory training can have positive effects on microstructure in older age. The degree of cognitive improvement was related to the degree of microstructural changes, demonstrating a relationship between behavioral and microstructural plasticity. No group differences or relationships between memory and WM microstructure were found in the young sample, indicating that microstructural plasticity in response to memory training is not necessarily larger in young adults. Plastic responses may depend on whether the nature of the training exceeds the pre‐existing range of processing capacity [Lövdén et al., 2010a] or induces a considerable change in environment. Thus, in this study, the training may have imposed a larger environmental change and challenge for the older adults relative to the young adults.

### Memory Improvement

We have recently shown specific training effects on memory improvement in older adults [de Lange et al., 2016]. The present results showed that within the respective age groups, both the young and the older training groups improved their memory to a larger extent than the active and passive controls. Thus, specificity of memory improvement was found across age. In this study, the number of tasks, group meetings, and contact with staff were matched between the training group and the active control group, controlling for the possible effect of these factors on memory performance. Furthermore, test sessions and time intervals were held identical for all participants to ensure that test–retest effects would not differ across groups. The inclusion of an active control group in this study strengthens the validity by allowing comparison of effects related to general components of the participation, and effects related to the specific components of the memory training [Hart et al., 2008; Law et al., 2014].

In accordance with previous studies, both the young and the older training groups improved their memory performance considerably in response to the training [Jones et al., 2006; Nyberg et al., 2003]. While some studies have demonstrated similar performance gains in young and older adults when individual differences in baseline performance are controlled for [Carretti et al., 2007], our results showed that the young adults improved their memory to a larger extent than the older adults when controlling for individual differences in baseline performance. Thus, the results align with training studies showing age differences in cognitive improvement [Baltes et al., 1992; Brehmer et al., 2012; Burki et al., 2014; Dahlin et al., 2008]. Some studies indicate that older adults may not be able to utilize mnemonic techniques as efficiently as young adults, due to age‐related decline in general processing resources such as executive functions and processing speed [Jones et al., 2006]. Mnemonic techniques also rely on the ability to generate and manipulate mental images, which is known to decline with advancing age [Palermo et al., 2016]. Moreover, evidence suggests that older adults experience greater challenges related to comprehension and compliance in training studies. One study showed that 22% of the older participants failed to comply with instructions in memory strategy training [Verhaeghen and Marcoen, 1996]. However, this figure decreased substantially when the participants were given more time for encoding. Hence, the options for individual adjustments in encoding time, in addition to the repetition of instructions in the group sessions, may have enhanced the comprehensibility of the training program. The majority of the participants (84.4% of the young adults and 72.1% of the older adults) improved their performance on recollection of words in a specific order, indicating the use of a mental travel route [Legge et al., 2012]. Although we did not include any objective measures of the utilization of MoL beyond the word list test, the test was designed to measure improvement in performance by the use of this particular strategy.

As the focus of this study was to investigate the relationships between training gain and WM microstructure, transfer effects in terms of improvement on untrained tasks were not assessed. Although the evidence for transfer effects after strategy training is limited [Bailey et al., 2014; Ball et al., 2002; Derwinger et al., 2003; Jones et al., 2006; Neely and Bäckman, 1993], some studies have reported improvement on untrained tasks in both young and older adults after strategy training [Carretti et al., 2007; Cavallini et al., 2010; Vranić et al., 2013] and episodic memory training [Schmiedek et al., 2010]. One study showed larger transfer from strategy training when instructions about applicability were provided [Cavallini et al., 2010]. Thus, although beyond the scope of this article, the generalizability of training effects are not yet fully understood, and may represent a key area for future research. Importantly, the present findings support that both young and older adults can benefit from memory strategy training and that such training can affect WM microstructure in older adults, which is discussed in the next section.

### Group Differences in Microstructural Changes

In accordance with previous studies showing positive effects of cognitive training on WM microstructure in older adults [Bennett et al., 2011; Engvig et al., 2012; Lövdén et al., 2010b], the older training group showed less decrease in FA and a smaller increase in MD, RD, and AD compared to the control groups, indicating that the training had a positive impact on microstructural decline.

The predominant findings from cross‐sectional and longitudinal aging studies are decreased FA accompanied by increased MD, RD, and AD with older age [Barrick et al., 2010; Bender et al., 2015; Bennett et al., 2010; Burzynska et al., 2010; Charlton et al., 2010; Davis et al., 2009; Salami et al., 2012; Salat et al., 2005; Sexton et al., 2014; Westlye et al., 2010]. As the group differences were partly driven by the age‐related decline in the control group, it is likely that the memory training may serve as a maintaining factor for WM microstructure in older age [Engvig et al., 2012].

Changes in both FA and RD have been associated with myelination in animal studies [Blumenfeld‐Katzir et al., 2011; Song et al., 2005]. Thus, the group differences observed on these metrics could be driven by differential changes in myelination. Increased immunofluorescence staining of myelin basic protein (MBP), which is indicative of myelination, has been observed in animals in co‐occurrence with increased FA after training [Blumenfeld‐Katzir et al., 2011; Sampaio‐Baptista et al., 2013]. However, as axonal membranes also contribute to anisotropic diffusion [Beaulieu, 2002], the observed differences in FA change may have been influenced by the condition of axonal membranes. The group differences in general diffusivity reflected by MD may indicate differential changes in relatively isotropic structures such as astrocytes. Animal studies have shown changes in the activation of astrocytes as an effect of spatial memory training [Blumenfeld‐Katzir et al., 2011; Sagi et al., 2012], which may underlie reductions in MD through intracellular to extracellular ratio alterations or cellular tissue swelling [Le Bihan et al., 2001; Theodosis et al., 2008]. However, myelination of axons in crossing fiber regions may also influence MD [Mackey et al., 2012], thus, the interpretation of the underlying changes in DTI metrics depends on the local fiber architecture. Although evidence suggests that DTI may be sensitive to underlying cellular changes with sufficient volumetric contribution [Fields, 2015; Sagi et al., 2012], the signal changes require careful interpretation as the neurobiological underpinnings cannot be directly inferred [Wheeler‐Kingshott and Cercignani, 2009; Zatorre et al., 2012]. Although the signal may be modulated by cellular properties and myelination, it is also influenced by how axons are laid out within the voxel, as the gradient is applied along a given axis [Jones et al., 2013].

The group differences in WM microstructure were found in areas overlapping the corpus callosum, the corticospinal tract, the cingulum bundle, the superior longitudinal fasciculus, and the anterior thalamic radiation. The cognitive processes involved in mnemonic strategy training are likely to rely on multiple brain areas. Thus, the highlighted areas may represent regions of importance for efficient information transfer that is beneficial for cognitive gains after this type of training. However, although individual studies have shown relationships between cognitive processes and WM properties in highly specific regions [Kerchner et al., 2012; Zhu et al., 2015], the overall evidence does not demonstrate a high degree of regional specificity in the relationship between WM microstructure and cognition [de Lange et al., 2016; Madden et al., 2009; Salthouse, 2011].

The training affected WM microstructure in the older adults only. Although this was unexpected, the finding supports other studies showing that plastic responses to cognitive training are not necessarily larger in young adults relative to older adults [Kempermann et al., 2002; Lövdén et al., 2010b). In a theoretical framework suggested by Lövdén et al. [2010a], plastic alterations in brain and behavior are thought to take place when there is a mismatch between the functional capacity and the environmental demands. The capacity for variations in behavior that do not require structural brain changes is referred to as flexibility. Flexibility can generate improvements in performance, but does not require changes in intrinsic capacity as opposed to plasticity [Noack et al., 2009]. Thus, flexibility depends on the pre‐existing range of processing capacity, while plasticity takes place only when the demands placed exceed the existing capacity. In view of this theory, the results may indicate that the memory training more substantially exceeded the functional capacity of the older adults, thus resulting in microstructural alterations solely in this group. Although the training posed increasing demands and the individuals could adjust the tasks to their own level, the intervention itself may not have provided demands as substantially exceeding the functional capacity in young adults, which is considered to be crucial for the initiation of plastic responses [Lövdén et al., 2010a].

It should be emphasized, however, that there was a cognitive change × age interaction, showing that the younger adults still managed to improve their memory performance more than the older adults. Hence, the observed divergence of behavioral and brain responses to training with age may be interpreted within a supply–demand framework. The improvement of performance in response to environmental demands may have been within the functional capacity of the young adults, while the demands may have exceeded the functional capacity of the older adults, thus, even more modest improvements would require brain changes. This is in line with the nature of the training being likely to have imposed a larger overall change in environment for the older adults. Indeed, the younger adults were in a phase where memory training may be more intrinsic to their everyday life, with studies and new work tasks typically posing continuous demands. The training was thus more likely to represent a considerable environmental change for the group of older adults, of which the majority of the individuals were retired. Thus, microstructural plasticity in response to memory training may depend on whether the level of the training exceeds the pre‐existing range of processing capacity [Lövdén et al., 2010a], and whether the nature of the training induces a considerable change in the environment.

### Relationships Between Memory Improvement and Microstructural Changes

Only few studies have documented relationships between cognitive improvement and altered WM microstructure in older adults [Bennett et al., 2011; Engvig et al., 2012]. Our results showed that the degree of cognitive benefit from memory training was related to the degree of change in WM microstructure in the older adults. Thus, the older participants who improved their memory performance to the largest extent showed a decrease in MD, RD, and AD, and an increase in FA, suggesting that these microstructural changes may be part of a neurobiological substrate for the behavioral improvements.

Importantly, the follow‐up analyses suggested that these relationships varied depending on how training gain was measured, that is, difference scores or standardized residuals. Difference scores are commonly used as a measure of training outcome [Engvig et al., 2010; Lövdén et al., 2010b], as is absolute scores, that is, performance after training [Draganski et al., 2004] and proportional gain such as percentage scores [Engvig et al., 2014]. Absolute scores and difference scores may be suitable as a way of measuring change, but do not take into account differences in relative improvement across individuals. Thus, difference scores do not account for the influence of baseline variance in analyses. For instance, two individuals—one with a low baseline score (5 points) and one with a high baseline score (10 points)—may both exhibit the same training gain (2 points). In a difference analysis, they are treated equivalently, even though their gains relative to baseline (40% and 20%, respectively) are not equal. Standardized residuals, however, provide a measure of training gain where baseline performance is accounted for. Starting point has been shown to influence the potential for cognitive improvement with training, both in terms of performance level at baseline [Lövdén et al., 2012a; Sandberg et al., 2015] and in terms of microstructural brain characteristics [de Lange et al., 2016]. A follow‐up partial correlation analysis (corrected for age and sex) showed that degree of memory improvement was related to baseline performance on both CVLT learning (*r* = 0.436, *P* = 0.004) and CVLT recall (*r* = 0.507, *P* = 0.001) in the older sample. No such relationships were found in the young sample. However, as individual starting point is likely to influence training gain [Lövdén et al., 2012a], baseline variance should in general be taken into account in training studies.

The lack of relationships between memory improvement and change in WM microstructure in the young adults may indicate that memory improvement in this group did not require microstructural plastic alterations. The young adults completed a lower number of tasks during the training period relative to the older adults. However, in correspondence with other studies that have failed to observe an association between the amount of time spent on the training and plastic responses [Boyke et al., 2008; Driemeyer et al., 2008], the results showed that number of tasks completed did not relate to either memory improvement or microstructural plasticity.

## Conclusions

This study provides evidence of relationships between microstructural alterations and cognitive improvement after memory training in older relative to young adults, and demonstrates that both cognitive and microstructural plasticity is preserved into older age. The somewhat counterintuitive lack of microstructural changes in the young group may imply that the demands of the memory training, despite being dynamically adapted to performance levels, did not exceed their existing range of processing capacity, and thus did not require microstructural plasticity [Lövdén et al., 2010a). Hence, a matched training program adapted to individual performance level for young and older adults may promote specific cognitive improvements for all, yet fail to promote structural plastic alterations in both age groups, due to age‐related differences in flexibility, or perhaps the greater overall changes in experience and environment for older adults. Further investigations are required to determine whether this also applies to other brain characteristics and how the changes develop over extended time periods. As the study protocol includes impending long‐term follow‐up, future research will focus on assessing whether the reported training effects are transient or maintained over time.
